# Do Gross and Fine Motor Skills Differentially Contribute to Language Outcomes? A Systematic Review

**DOI:** 10.3389/fpsyg.2019.02670

**Published:** 2019-12-03

**Authors:** Sandy L. Gonzalez, Veronica Alvarez, Eliza L. Nelson

**Affiliations:** ^1^Department of Psychology, Florida International University, Miami, FL, United States; ^2^Department of Applied Psychology, New York University, New York, NY, United States

**Keywords:** motor, fine motor, gross motor, language, infancy, toddlerhood, preschool

## Abstract

**Background:** Changes in motor development provide children with new learning opportunities to interact with objects, their environment, and with caregivers. Previous research finds that both gross and fine motor skills are predictive of later language outcomes across early infancy and childhood. However, gross and fine motor skills afford different types of interactions. Thus, gross and fine motor skills may potentially differ in the developmental trajectories through which cascading changes in language may occur. The aim of the present study was to investigate whether there are differences in the predictive capacities of gross and fine motor skills toward language outcomes across infancy and early childhood in typical development.

**Method:** A systematic review of existing literature on motor-language cascades was conducted in across studies measuring gross and/or fine motor and language development in children from 0 to 5 years old. Searches were conducted in PsycINFO, PubMed, and MEDLINE. Keywords used were a combination of “gross motor,” “fine motor,” “motor performance,” “motor development,” or “psychomotor development” along with “language,” “language development,” or “communication skills.” Two independent reviewers screened abstracts and full texts based on inclusion and exclusion criteria.

**Results:** A total of 23 articles were retained. Of these, seven studies measured only gross motor skills, four studies measured only fine motor skills, and 12 studies measured both gross and fine motor skills in the same study. Studies used a variety of measures to assess gross motor skills, fine motor skills, and language development (e.g., parent report, in lab observations, standardized assessment), and findings varied based on analyses used. Results demonstrated that both gross and fine motor skills are related to language outcomes, but due to a smaller amount of studies testing fine motor skills, conclusions regarding whether one is more important for language outcomes cannot be drawn.

**Conclusions:** We conclude that both gross and fine motor skills help foster language development from infancy to early childhood. Limitations regarding current knowledge regarding the mechanisms that underlie motor-language cascades are discussed, as well as the need for more studies on fine motor skills.

## Introduction

Motor development research has previously been considered the Cinderella of developmental science: central to children's experiences, but rarely in the spotlight (Rosenbaum, 2005; Adolph et al., 2010). A historically maturational approach to motor skills was predominant in the early twentieth century, which mainly argued that motor development unfolds via predetermined biological changes, with little to no intervention from environmental or cognitive domains (e.g., Gesell and Amatruda, 1945). Isolation of motor skill from cognition resulted in very little research focusing on the role of motor skills, instrumental to infant independence and exploration, on other domains of development such as language. Similarly, views of language as modular and universal (Chomsky, 1975) likely also contributed to further divorcing motor skills and language. However, continuing shifts toward ecological and systems approaches to development have allowed recent research to embrace the possibility of cross domain interactions resulting in cascading changes throughout periods when the developing system is in flux (Gibson, 1988; Thelen and Smith, 2006; Masten and Cicchetti, 2010; Spencer et al., 2011). In the burgeoning literature on motor-language cascades, increasingly more research finds that motor skills matter for children's language outcomes (e.g., Iverson, 2010; Oudgenoeg-Paz et al., 2012; Walle, 2016).

Motor development is often broadly divided into gross motor and fine motor skills. Gross motor skills pertain to skills involving large muscle movements, such as independent sitting, crawling, walking, or running. Fine motor skills involve use of smaller muscles, such as grasping, object manipulation, or drawing. While many studies have investigated the role of motor skills on language development (e.g., Walle and Campos, 2014; Leonard et al., 2015; Choi et al., 2018), it is unclear whether one type of motor skill is more consistently related to language outcomes then the other. Recent research highlights that delays in motor development are linked to diagnoses such as Autism Spectrum Disorder and Specific Language Impairment (Leonard and Hill, 2014; West, 2018). Specifically, motor issues can be seen early on in at risk populations, prior to diagnosis, positioning motor skills as a potential early marker for later outcomes (Bhat et al., 2012; Flanagan et al., 2012; Lebarton and Iverson, 2013; Libertus et al., 2014). It is important to note that motor development is neither sufficient nor necessary for language development, as not all individuals with motor issues may present adverse language development (Iverson, 2010). However, given recent findings indicating that motor skills are part of a host of factors co-acting on language development, it is pertinent that researchers investigate potential differences in how motor skill types relate to language development in typical samples to inform further research in clinical settings.

Thus, the current systematic review will discuss existing literature on gross and fine motor skills in relation to language outcomes, and will focus on disentangling the cross relations between language development and gross and fine motor skills. We will focus on infancy through early childhood (0–5 years of age) in order to capture findings during early development, as both motor skills and language abilities are rapidly changing during this time period, allowing for a better understanding of how motor and language relate while the system is in flux (Thelen and Smith, 2006; Masten and Cicchetti, 2010).

## Methods

### Study Design

A systematic review was conducted on existing literature spanning infancy through early childhood on the cascading relations between motor and language development using PRISMA guidelines.

### Search Strategy

Article searches across the following databases were conducted: PsycINFO, PubMed, and MEDLINE beginning on July, 6th, 2018. Searches on Google Scholar were not conducted in order to avoid potentially personalized search results (Holone, 2016; Curkovic, 2019). Keywords used were a combination of “gross motor,” “fine motor,” “motor performance,” “motor development,” or “psychomotor development” along with “language,” “language development,” or “communication skills.” When available, database options for peer-reviewed articles only, human, and age limits of participants (infancy through 5 years old) were selected to better tailor search results for the focus of the current review. A total of 6,210 articles were identified as potentially relevant.

Two independent reviewers (the first and second author) further screened abstracts using the online program Abstrackr, an open-source tool for systematic reviews (Wallace et al., 2012). Abstrackr presents potentially more relevant articles early on during the abstract review process, and allows for semi-automated abstract rejection through use of algorithm based machine learned patterns that utilize the patterns of prior manual abstract rejections by the human reviewers (Rathbone et al., 2015). Research demonstrates that the Abstrackr algorithm has good precision with low levels of false-negatives depending on the complexity of the systematic review (Rathbone et al., 2015). Therefore, additional tools such as Google Scholar were not used during the search phase. In order to maximize accuracy of the Abstrackr algorithm while balancing expediency, both independent reviewers screened 3,000 abstracts manually, and the remaining 3,210 abstracts were screened utilizing the Abstrackr algorithm. Of the 3,210 remaining abstracts screened exclusively by the Abstrackr algorithm, two were tagged as potentially relevant for further full text review. Abstract review on Abstrackr was inclusive of duplicates. Among the full sample of 6,210 articles, 2,049 were identified as duplicates and were removed from further full text review after abstract screening. Two additional articles were added by the first author based on prior knowledge of their relevance to the systematic review, and one article was added based on reviewer suggestions, for a total of 129 articles selected for full text review.

### Eligibility Criteria

Abstracts were screened using the following inclusion criteria: (1) studies that included a typically developing sample in order to not reproduce other existing reviews/meta analyses on atypical development (e.g., West, 2018), (2) studies with a sample within the range of 0 to 5 years of age, (3) studies that measured both motor and language skills, and (4) studies reported in English. Exclusion criteria included: (1) case studies, (2) studies with only atypical populations, (3) studies where only motor or only language skills were measured and results were only suggestive of motor-language links, (4) studies that did not differentiate gross and fine motor skills (e.g., had one global motor score), (5) studies where the measured motor skills were exclusively speech-motor/oro-motor control (to avoid conflating with measures of language), rhythmic arm movement, handedness, gesture, motor imitation, or synchronized finger tapping (to limit our review to general gross or fine motor skill abilities, rather than facets of skill execution), (6) studies where language skills were only measured based on babbling or vocalizations/pre-vocal behaviors. If it was unclear whether a study met inclusion or exclusion criteria based on the abstract alone, the reviewers discussed the abstract together. If an agreement could not be made between reviewers based on the abstract alone, the article was included for further full text review.

**Figure 1:**
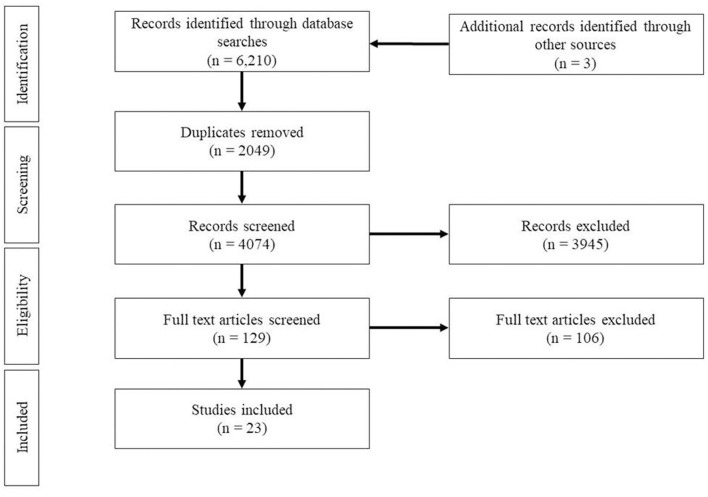
PRISMA flow diagram of study selection process.

Full text review was conducted by the first and second author, with any disagreements/final decisions regarding inclusion and exclusion discussed among all three authors when necessary. The criteria discussed above continued to be implemented during full text review. Articles were thoroughly read for inclusion of analyses that detailed motor-language cascades in typical samples, as studies with an atypical focus often included control groups which passed inclusion criteria during abstract review, but upon full text reading (1) did not conduct analyses on motor-language cascades with the typically developing samples (i.e., conducted typical vs. atypical group comparisons only, or did not measure motor or language skills in the typical sample), or (2) grouped atypical and typical samples for power purposes for motor-language cascade analyses which did not allow for reporting of typical results alone. Only studies in which clear results for typically developing children were reported were included for final article inclusion. Studies which included children 0–5 years, but also included older age ranges were only included if results for ages from 0 to 5 years were reported separately from the full sample and if motor and language results were both measured at a time point between 0 and 5 years old. The PRISMA flow chart (Figure 1) indicates how many full text articles were excluded.

### Prevention of Bias and Quality Assessment

In an effort to reduce bias, abstracts and articles were screened by two independent screeners. Training on how to use Abstrackr was conducted using tools available through the Abstrackr website prior to any screening. Both authors also practiced scoring a subset of articles together prior to independent screening, and discussed the thought process behind inclusion and exclusion decisions during the training period. Abstrackr allows users to keep track of disagreements between the two reviewers. Thus, at a half way point during independent screening; the two independent screeners discussed existing conflicts flagged by Abstrackr in order to adjust all further abstract screening accordingly. Disagreements were settled via discussion. Moreover, in an effort to further reduce bias, the authors included results from typically developing control samples reported in studies focused on atypical development. This strategy was done in order to reduce bias toward reporting of only “positive results,” which was more likely with studies that solely focused on typical samples.

All articles selected for final inclusion in the current systematic review were assessed for quality based on Downes et al. (2016) Appraisal tool for Cross-Sectional Studies (AXIS) tool. Quality assessment with AXIS is based on 20 questions regarding inclusion or exclusion of information in the introduction, methods, results, and discussion. The original AXIS measure does not provide a numerical score. However, all studies received scores positive scores for more than half of the items on AXIS. No studies were excluded based on quality assessment. Results are detailed in Supplementary Table 1.

## Results

### Synthesis

At total of 23 peer-reviewed articles were included in the current systematic review (Wolff and Wolff, 1972; Butterworth and Morissette, 1996; Lyytinen et al., 2001; Alcock and Krawczyk, 2010; Iverson and Braddock, 2010; Rhemtulla and Tucker-Drob, 2011; Oudgenoeg-Paz et al., 2012, 2015, 2016; Karasik et al., 2014; Muluk et al., 2014, 2016; Suggate and Stoeger, 2014; Walle and Campos, 2014; Wang et al., 2014; He et al., 2015; Leonard et al., 2015; Libertus and Violi, 2016; Walle, 2016; West et al., 2017; Choi et al., 2018). Information was extracted regarding the main purpose, study design, sample size, ages tested, measures used to test gross and/or fine motor, measures used to measure language. All extracted information can be found in Table 1. Studies included in the present systematic review spanned 1972 to 2018, with the most publications occurring in 2016 (*n* = 5). The majority of studies used longitudinal methods (*n* = 11), with nine studies using cross sectional methods. One study had multiple studies and used both longitudinal and cross-sectional methods (Walle and Campos, 2014), and two studies used longitudinal methods, but results reported in this systematic review only pertain to cross-sectional results at one age as the studies also included older ages and analyses allowed for reporting results only for the ages of interest to this systematic review (Rhemtulla and Tucker-Drob, 2011; Cameron et al., 2012). Sample size varied across studies ranging from 16 to 11,999 (sample sizes reported refer only to number of typically developing children). Overall, 17 studies focused solely on typically developing children, while six studies included both typical and atypical developing samples.

**Table 1:** Articles included in systematic review.

**Study**	**Study design**	***N*^a^**	**Ages tested^b^**	**Motor assessment**	**Language assessment**
Alcock and Krawczyk (2010)	CS	129	21 m/o	GM and FM: BSID or ASQ items	MCDI (UK)
Butterworth and Morissette (1996)	LG	27	8.5–14.5 m/o (monthly assessments)	FM: Pincer grip (4 trials)	MCDI
Cameron et al. (2012)	CS^c^	213	3–5 y/o	GM and FM: Early Screening Inventory –Revised	Woodcock Johnson- Picture Vocabulary
Choi et al. (2018)	LG	69	Motor: 6–24 m/o (assessments every 6 months) Language: 36 m/o	GM and FM: MSEL	MSEL
He et al. (2015)	CS	US sample: 40 Chinese sample: 42	US sample: 12.5 m/o Chinese sample: 13–14.5 m/o	GM: Parent reported age of crawling or walking onset	MCDI (US and Mandarin)
Houwen et al. (2016)	CS	130	0–3 y/o	GM and FM: BSID (Netherlands)	BSDI (Netherlands)
Iverson and Braddock (2010)	CS	16	3–5 y/o	FM: CDI and Battelle Developmental Screening Inventory	PLS and measures from in lab observation
Karasik et al. (2014)	CS	50	13 m/o	GM: experimenter verified crawling vs. walking status	MCDI
Leonard et al. (2015)	LG	55	Motor: 7 m/o Language: 14, 24, and 36 m/o	GM and FM: MSEL	VABS
Libertus and Violi (2016)	LG	29	Motor: 3–5 m/o (8 weekly assessments) Language: 10 and 14 m/o	GM: Sitting duration FM: Grasping duration	MCDI
Lyytinen et al. (2001)	LG	93	0–5 y/o	GM and FM: Parent reported milestones	MCDI
Muluk et al. (2014)	CS	347	3, 4, and 5 y/o	GM and FM: Denver Developmental screening items (Turkey)	Denver Developmental screening items (Turkey)
Muluk et al. (2016)	CS	505	6, 12, 18 and 24 m/o	GM: Denver Developmental screening Items (Turkey)	Denver Developmental screening items (Turkey)
Oudgenoeg-Paz et al. (2012)	LG	55	Motor: behavior onset^d^ Language: 6, 12, and 18 m/o	GM: Parent reported age of sitting or walking onset	MCDI (Netherlands)
Oudgenoeg-Paz et al. (2015)	LG	31	Motor: behavior onset^d^ and 20 m/o Language: 36 m/o	GM: Parent reported age of crawling or walking onset and observation of exploration through self-locomotion FM: Observation of object exploration	Spatial language
Oudgenoeg-Paz et al. (2016)	LG	59	Motor: behavior onset^d^ Language: 43 m/o	GM: Parent reported age of crawling or walking onset and observation of exploration through self-locomotion	PPVT (Netherlands), spatial language, and sentence repetition task
Rhemtulla and Tucker-Drob (2011)	CS^c^	8,950	4 y/o	GM: Assessed jumping, balancing, skipping, walking backwards, and catching a bean bag FM: Assessed building a gate with blocks, copying a square, triangle, and an asterisk	“Let's Tell Stories” oral language task
Suggate and Stoeger (2014)	CS	76	3–5 years	FM: Pegboard task, bead threading, and block turning	PPVT (German), body-object interaction words, manipulable words
Walle (2016)	LG	43	10–13.5 m/o (bi-weekly assessments)	GM: Parent reported age of crawling or walking onset	MCDI
Walle and Campos (2014)	LG/CS	LG: 44 CS: 75	LG: 10–13.5 m/o (bi-weekly assessments) CS: 12.5 m/o	GM: Parent reported age of crawling or walking onset	MCDI
Wang et al. (2014)	LG	11,999	3 and 5 y/o	GM and FM: ASQ	ASQ
West et al. (2017)	LG	25	2–19 m/o (bi-weekly assessments)	GM: Parent reported age of walking onset	MCDI
Wolff and Wolff (1972)	CS	55	4 and 5 y/o	GM and FM: Teacher report	Teacher report

*CS, cross-sectional; LG, longitudinal; m/o, months old; y/o, years old; GM, gross motor; FM, fine motor; BSID, Bayley Scales of Infant Development; ASQ, Ages and Stages Questionnaire; MSEL, Mullen Scales of Early Learning; CDI, Child Development Inventory; MCDI, MacArthur-Bates Communicative Development Inventory; PLS, Preschool Language Scales; VABS, Vineland Adaptive Behavior Scales; PPVT, Peabody Picture Vocabulary Test*.

a *Sample sizes reported include only typically developing children*.

b *Ages reported for systematic review include only ages of interest, full study included older ages*.

c *Results reported for systematic review are cross-sectional, full study is longitudinal*.

d *Exact ages not reported given variability in onset ages*.

In terms of measurement, 12 studies assessed both gross motor skills and fine motor skills for motor-language analyses. However one study by Muluk et al. (2016) did not provide clear results for fine motor skills, and thus only gross motor results are discussed in this review. Seven studies measured only gross motor skills, and four studies measured only fine motor. Studies used a variety of assessment types to measure motor skill. Studies measuring gross motor skill most frequently used parent reported age of skill acquisition (*n* = 6), while studies measuring fine motor skill used in lab tasks/observations (*n* = 6). In terms of language, most studies on measuring fine motor skills used a parent report measure for language skills (*n* = 11; e.g., MacArthur Bates Communicative Development Inventories, Ages and Stages Questionnaire). Studies measuring gross motor skills also largely used parent report for language skills (*n* = 11). Although studies were not selected based on measures that differentiated between receptive and expressive language skills, the majority of studies measured both receptive and expressive skills separately (*n* = 12). Additionally, two studies measured language skills related to words relevant to actions (e.g., spatial words, word related to high levels of body interaction) in addition to other language measures, and one study only measured production of spatial language.

### Gross Motor Skills Results

Results for this section will first detail the relation between gross motor and language skills, categorized by ages studied and study methodology (cross-sectional vs. longitudinal). At the end of this section commonalities across gross motor studies will then be discussed.

#### Cross-Sectional Studies With Infants and Toddlers

Six articles measured the relation between gross motor skills and language development utilizing cross-sectional methods in infants and toddlers (Alcock and Krawczyk, 2010; Karasik et al., 2014; Walle and Campos, 2014; He et al., 2015; Houwen et al., 2016; Muluk et al., 2016). Overall, the studies reviewed in detail below do find concurrent relations between gross motor skills and language development within U.S., U.K., Chinese, Turkish, and Dutch samples of infants. However, for two of the six studies, accounting for additional covariates such as cognitive skills or other motor skills and demographic variables, reduces gross motor's significant contribution to language (Alcock and Krawczyk, 2010; Houwen et al., 2016). Studies have used a variety of methods to operationalize “gross motor”: two studies used parent reported walking onset exclusively (Walle and Campos, 2014; He et al., 2015), one study used both standardized assessment and parent questionnaires (Alcock and Krawczyk, 2010), two studies utilized a standardized assessment or items derived from a standardized assessment (Houwen et al., 2016; Muluk et al., 2016), and one study used experimenter observation of crawling or walking (Karasik et al., 2014). Most (four out of six) relied on parent report for measures of language skill (Alcock and Krawczyk, 2010; Karasik et al., 2014; Walle and Campos, 2014; He et al., 2015). Overall, 50% of studies in this section suggest that gross motor and language skills are related concurrently in infancy, particularly when assessing gross motor skills from a single behavior (e.g., walking) rather than a global gross motor score.

Using a wide cross-sectional sample spanning 3 months to 3 years of age, Houwen et al. (2016) measured gross motor skill and language using the Dutch Bayley Scales of Infant Development (BSID) which includes subscales for gross motor skills and expressive and receptive language. Gross motor scores were significantly positively correlated with both expressive and receptive communication scores, however this relation did not hold once controlling for cognitive level. Focusing on a sample of Turkish children, Muluk et al. (2016) measured gross motor skills and language ability using a cross-sectional sample of children at 6, 12, 18, and 24 months of age. Gross motor skills and receptive and expressive language skills were measured using individual items form the Denver Developmental Screening standardized for use with Turkish children. Items used for gross motor and language varied across age groups. At 6 months, the “pull to sit (no head lag)” item was positively significantly correlated to the language item “turns to sound.” Infant's ability to “lift chest with arm support” was also significantly positively correlated to the language item “turns to voice” at 6 months. Both of these 6 month relations were significant when controlling for each other along with various covariates (sex, SES, maternal education, and “working for a toy out of reach”). At 12 months, being able to “stand holding on” was positively significantly related to the language item “mama/dada specific” and to being able to “say 4 words other than mama/dada.” The item “stands alone for 10 seconds” was also positively significantly correlated to being able “to say 4 words other than mama/dada.” These 12 month relations were significant when controlling for each other along with other covariates (SES, maternal age, and indicates needs not crying). At 18 months, the ability to “throw a ball” was significantly negatively correlated with “saying 4 words other than mama/dada,” while controlling for sex as a covariate. No results were reported for gross motor and language at 24 months.

Investigating motor and language development at 12 months of age, Walle and Campos (2014) measured the relation between quality of locomotion and language comparing same aged crawlers and walkers in a cross-sectional sample. Results indicated that walking infants had larger receptive and expressive vocabularies as measured via parent report on the MacArthur Bates Commutative Developmental Inventory: Words and Gestures (MCDI: WG) short form. He et al. (2015) reproduced these results in a cross-cultural study comparing U.S. and Chinese infants, with findings demonstrating that for both U.S. infants (about 12.5 months old) and for Chinese infants (between 13 and 14.5 months old), walkers demonstrated significantly greater receptive and expressive vocabulary, in English and Mandarin, respectively, compared to crawlers. When accounting for U.S. infants self-produced locomotion experience, walking status only marginally predicted receptive vocabulary, but a continued significant relation between walking status and expressive vocabulary remained. In Chinese infants, walking status continued to significantly predict both receptive and expressive vocabulary even when controlling for self-produced locomotion experience. When focusing specifically on receptive and expressive vocabulary for nouns, U.S. and Chinese infants who could walk both had larger noun and non-noun vocabularies compared to crawlers. However, the proportion of nouns to non-nouns for both receptive and expressive vocabulary was not significantly different between walkers and crawlers, indicating locomotor status did not matter in this case for U.S. infants. For Chinese infants, the proportion of nouns to non-nouns for receptive was not significantly different between walkers and crawlers, but the proportion of nouns to non-nouns for expressive language did significantly differ, indicating that Chinese children who could walk were likely to know more nouns than non-nouns in Mandarin than crawlers.

Comparably, Karasik et al. (2014) assessed differences in 13-month-old crawlers and walkers vocabulary size as part of a study on infant bidding styles. Crawling and walking status was determined from experimenter observation of the skill, and receptive and expressive vocabulary was measured using the MCDI. Results indicated that there was no significant difference in receptive or expressive vocabulary size between same-aged walkers and crawlers.

At 21 months of age, Alcock and Krawczyk (2010) measured gross motor skills using the BSID or with a questionnaire that was adapted to include gross motor questions from the Ages and Stages Questionnaire (ASQ) parent report measure. Language skills were measured using the Oxford MCDI, with additional questions about word combinations and grammatical usage (e.g., complexity) from the U.S. English MCDI: Words and Sentences (MCDI: WS). For infants with parent reported gross motor scores via questionnaire, gross motor skills were significantly positively correlated to receptive and expressive vocabulary, but not complexity. When utilizing standardized scores to combine infants who completed the BSID or the gross motor questionnaire, gross motor skills were not significantly correlated with language comprehension, production, or complexity. Standardized gross motor scores and questionnaire gross motor scores did not significantly predict receptive, expressive vocabulary, or complexity when accounting for oral motor movement, fine motor score, gesture, and symbolic gesture. Alcock and Krawczyk (2010) also examined motor-language relations in a subsample of infants who did not complete the oral motor test, but who did have gross and fine motor scores, in order to test the relation between upper and lower limb motor control and language. Results indicated that gross motor skill based on parent report did predict vocabulary production, but did not predict language comprehension or complexity, while controlling for fine motor score, gesture, and symbolic gesture.

#### Longitudinal Studies With Infants and Toddlers

A total of nine articles investigated the longitudinal relations between gross motor skills and language development (Lyytinen et al., 2001; Oudgenoeg-Paz et al., 2012, 2015, 2016; Walle and Campos, 2014; Leonard et al., 2015; Libertus and Violi, 2016; Walle, 2016; West et al., 2017). Longitudinal methods help inform researchers about length of cascading effects, and can provide knowledge regarding growth over time for both motor and language development. In this subset of longitudinal articles, eight out of nine articles (about 89%) demonstrate that gross motor skills are related to language skills (Lyytinen et al., 2001; Oudgenoeg-Paz et al., 2012, 2015, 2016; Walle and Campos, 2014; Libertus and Violi, 2016; Walle, 2016; West et al., 2017). Importantly, because longitudinal studies can provide information about skills over time, results here begin to show that the length of certain motor to language relations may change over time, and the contributions of motor to language may depend on skill type (e.g., Oudgenoeg-Paz et al., 2015). This portion of the literature also expands beyond parent reported onset of locomotion (i.e., crawling vs. walking) and begins to report on motor-language relations pertaining to behaviors such as sitting and locomotor exploration (Oudgenoeg-Paz et al., 2015, 2016; Libertus and Violi, 2016). Samples reviewed here included Dutch, Finnish, U.K., and U.S. infants. A total of 6 studies included covariates when analyzing gross motor to language relations. Results from these studies indicated that gross motor skills predicted language outcomes above and beyond age, concurrent motor abilities, and parent based social factors such as parent initiated joint engagement and viewing the infant as an individual (e.g., Libertus and Violi, 2016; Walle, 2016; West et al., 2017). Similarly to the cross-sectional studies reported in the previous section, existing literature supports the idea that gross motor skills play an important role in language development across infancy and toddlerhood.

Using video conferencing technology to measure infant sitting in the home, Libertus and Violi (2016) calculated growth in sitting skill (i.e., duration in independent sitting) over time from 3 to 5 months of age. Language skill was measured using the MCDI: WG later at 10 and 14 months old. Greater growth in duration of sitting was significantly positively related to receptive vocabulary at 10 and 14 months of age, even when including concurrent general motor skills as a covariate. In a study on the longitudinal relations between motor and language in typically developing infants and infants at high-risk for autism, Leonard et al. (2015) assessed gross motor skills at 7 months using the gross motor subscale of the Mullen Scales of Early Development (MSEL). Language skill was measured at 7, 14, 24, and 36 months using the Vineland Adaptive Behavior Scales (VABS). Results accounted for visual receptive skill at 7 months, and found that for the typically developing sample gross motor ability at 7 months was not predictive of growth in receptive of expressive language skills from 7 to 36 months.

In another study focused on predicting language growth from early gross motor skills, Oudgenoeg-Paz et al. (2012) found that age at which independent sitting was attained significantly predicted productive language skill (as measured by the Dutch short form versions of the MCDI) at 20 months, with younger sitting age predicting greater productive vocabulary. Age of independent walking significantly predicted rate of expressive vocabulary growth from 16 to 28 months, with younger walking age predicting greater language growth. Age of independent walking did not predict language skill at 20 months, and age of sitting did not predict language growth. Expanding on these results, Oudgenoeg-Paz et al. (2015) measured spatial language production at 36 months using interactive assessments during home visits. In addition to utilizing parent reported age of acquisition of sitting and walking, spatial exploration was also measured by trained observers during structured observation of infants' actions with two sets of objects assessing spatial-relational exploratory behavior and exploration through self-locomotion at 20 months. Results indicated that age of independent sitting did not significantly predict spatial language use, but age of walking acquisition did. Amount of exploration through self-locomotion was also significantly positively related to productive spatial language, while spatial-relational exploration was not related to spatial language. Importantly, exploration through self-locomotion partially mediated the relation between walking age and spatial language, indicating the effect of walking age on spatial vocabulary is partly explained by amount of self-locomotor exploration. Additional work by Oudgenoeg-Paz et al. (2016) measured general receptive vocabulary using the Peabody Picture Vocabulary Test (PPVT), grammatical and lexical categories during a sentence repetition task, and productive spatial language based on knowledge of locative prepositions and directional verbs during home visits at 42 months. Gross motor skills were assessed based on parent reported age of walking onset, and during a structured observation by trained observers of exploration through self-locomotion at 20 months during a home visit. Age of walking did not significantly predict spatial language. Exploration through self-locomotion completely significantly positively mediated the relation between walking age and spatial language. Walking age did not significantly predict receptive vocabulary or use of grammatical and lexical categories, and exploration through self-locomotion did not mediate any of these relations. Across these three studies, a more complex picture of motor-language cascades is seen for gross motor skills. Independent sitting is important for language outcomes, but with more time between sitting acquisition and when language is measured, it is likely that the cascading effects of sitting are no longer as strong, or that they are superseded by more novel skills (e.g., walking). But even in the case of walking, by 42 months there is no relation between age of walking onset and general vocabulary, although walking was predictive of language growth across earlier time points. Similarly, walking onset no longer was predictive of spatial language at 42 months, although it had been at 36 months. Instead, amount of self-locomotor exploration at 20 months predicted spatial language at 42 months.

As part of a larger longitudinal study, Lyytinen et al. (2001) compared typically developing infants and infants with children at risk for dyslexia. Gross motor skill was measured based on parent report of age of onset of gross motor milestones, with analyses using each infant's deviation from a calculated median growth curve based on gross motor skill attainment across various skills over the first year of life. Language development was measured using the MCDI: WG for receptive and expressive vocabulary at 12 and 14 months, and MCDI: Words and Sentences (MCDI: WS) for productive vocabulary at 24 and 30 months. For results specific only to typical children, gross motor skills were significantly positively correlated with vocabulary comprehension at 12 and 14 months, but not with vocabulary production at 14, 24 or 30 months. Focusing on changes in locomotion style over time in relation to language development, Walle and Campos (2014) longitudinally followed infants across the transition from crawling to walking. Specifically, gross motor skill was assessed using parent reported age of walking and crawling onset to calculate length of walking experience. Language was measured using the MCDI: WG to measure receptive and expressive vocabulary. Results indicated that walking experience was significantly predictive of receptive vocabulary size, with greater walking experience predicting larger receptive vocabulary. Significant increases in receptive vocabulary were seen at the transition from crawling to walking, and between walking onset and 2 weeks post walking onset. No significant increases in vocabulary were seen between 2 weeks after and 4 weeks after walking onset, or at 4 and 6 weeks of walking experience, or at 6 and 8 weeks of walking experience. For productive vocabulary, more walking experience significantly predicted greater expressive vocabulary. There was no significant increase in expressive vocabulary during the transition from crawling and walking. There was also no significant increase in expressive vocabulary between walking onset and 2 weeks post walking onset, or between 2 weeks after and 4 weeks after walking onset, or at 4 and 6 weeks of walking experience. A significant increase in expressive vocabulary was seen between 6 and 8 weeks post walking onset. Overall, results indicate that walking onset is correlated with immediate growth in receptive vocabulary, and also with later growth in expressive vocabulary.

Findings by Walle and Campos (2014) have spurred additional replications that further support the role of walking onset within language development. Results from Walle (2016) indicate that walking experience (calculated based on walking onset) was significantly positively predictive of receptive and productive vocabulary size (as measured by the MCDI: WG). Importantly, walking experience significantly predicted receptive and expressive vocabulary, even when controlling for parent initiated joint engagement, parent report of viewing the infant as an individual, and age. In a study comparing the effects of walking onset on language in typically developing infants and in infants at high risk for autism, West et al. (2017) followed infants longitudinally across the transition from crawling to walking, and found that both receptive and expressive vocabulary (as measured by the MCDI: WS) increased after infants final crawling visit and after walk onset while controlling for infant's age at the time of walk onset.

#### Cross-Sectional Studies Spanning Pre-kindergarten and Early Childhood

Expanding into preschool and early childhood age ranges, four studies investigated the role of gross motor skill on language development using cross-sectional methods and are reviewed in detail below (Wolff and Wolff, 1972; Rhemtulla and Tucker-Drob, 2011; Cameron et al., 2012; Muluk et al., 2014). The majority of the samples discussed in this section were of U.S. based children, with one study reporting on Turkish children (Muluk et al., 2014). In general, measures and methods in this section are mixed with two studies that utilized gross motor and language measures based performance on individual tasks (Rhemtulla and Tucker-Drob, 2011; Muluk et al., 2014), and two studies using global gross motor scores from assessments or questionnaires (Wolff and Wolff, 1972; Cameron et al., 2012). Novel to the review thus far, one article also opted to use teacher report for both gross motor and language skills (Wolff and Wolff, 1972). In general, use of such disparate measurements results in a limited understanding regarding gross motor skills at a global level, but highlights potential differences across individual skills beyond crawling or walking that were common in infant studies and their relation to language.

For the studies by Cameron et al. (2012) and Rhemtulla and Tucker-Drob (2011), both used longitudinal methods, however results reported in the current systematic review only include only ages 5 years or younger. Rhemtulla and Tucker-Drob (2011) provided cross-sectional correlations at 4 years of age which are reviewed below. Cameron et al. (2012) indicated in their results that the oldest child to complete a motor assessment at the beginning of their study (beginning of kindergarten) was 5.75 years old (69 months). Measurements at a second time point were described as being in spring of kindergarten, which indicates that the older children may have already turned six (72 months) by that time point. The only cross-sectional study within this age range that included covariates utilized backwards regression and reported only on the best fitting models per age group, which limits our interpretation of gross motor to language relations as covariates varied widely across ages and individual language measures (Muluk et al., 2014). At this age range, three studies (75%) reviewed support the idea that gross motor skills continue to be related to language outcomes concurrently, but we would argue that more recent and rigorous cross-sectional studies are required.

In a sample that includes 3, 4, and 5 year olds, Muluk et al. (2014) measured gross motor skills and receptive and expressive language skills using selected items from the Denver II for use in Turkey. Both gross motor and language measures varied in skills measured and number of items by age group. At 3 years, being able to “ride a tricycle” was significantly correlated to “comprehension of one preposition,” but did not hold significance when accounting for other covariates. The ability to “jump up” was significantly positively correlated to “use of plurals” and “comprehending one preposition,” and continued to be related to “comprehending one preposition” when accounting for other covariates. When accounting for other covariates, “jump up” was significantly related to and “gives first and last name” and being able to “define six words.” Balancing on one foot was also significantly positively correlated to using plurals and being able to give first and last name at 3 years, but was no longer related to these items after controlling for other covariates. When accounting for variability in other skills and factors, “balancing on one foot” was related to the language item “knowing one function.” At 3 years, being able to run was significantly negatively correlated to the language item “naming three pictures,” however this relation did not hold when accounting for other covariates. At 4 years “hopping on one foot” and “broad jumping” ability were not correlated to any language items, however hopping on one foot was related to knowledge of “how to use on object” once accounting for other covariates. At 5 years, “heel-to-toe walking” ability was significantly positively correlated to language items “defines six words” and “counting two blocks,” however none of these relations were maintained when accounting for other covariates.

In a similar study utilizing individual lab based items to measure gross motor and language skills, Rhemtulla and Tucker-Drob (2011) utilized longitudinal growth modeling methods across 3 to 7 years of age, but provide single time point data based on intercept values on motor language relations at 4 years of age. Gross motor skills were measured by experimenters during specific tasks: jumping, balancing, hopping, skipping, walking backwards, and catching a bean bag. Oral language skills were measured using the Lets Tell Stories task. Oral language skills at 4 years were significantly positively correlated to concurrent gross motor scores. In the Cameron et al. (2012) study on motor and executive function in relation to kindergarten achievement, motor skills were measured at the beginning using the Early Screening-Inventory-Revised, with analyses related to gross motor skills based on a composite score. Language production skills were assessed using the Woodcock Johnson Vocabulary subtest. Gross motor skills were not significantly correlated to language skills measured in the fall of kindergarten above and beyond fine motor skills, or other covariates such as executive functioning, age, sex, ethnicity, maternal education, or motor age.

In a departure from lab based or parent reported measures, Wolff and Wolff (1972) utilized teacher ratings on a Likert scale to measure both gross (e.g., degree to which the child is motorically active, degree to which she engages in gross bodily movements, etc.) and verbal language skills (e.g., verbal output and skill level). Gross motor skills were significantly positively related to verbal output scores, but not to verbal skill indicating that potentially at preschool age gross motor skills still related to quantity of language use (similar to some results from infancy and toddlerhood), but not to quality.

#### Longitudinal Studies Spanning Pre-kindergarten and Early Childhood

One study measured the relation between gross motor and language development across preschool and early childhood (Wang et al., 2014). Based on the one study reviewed below, results indicate that in this age range gross motor skills continue to predict language outcomes, but not as consistently longitudinally as seen in infancy and childhood. In general, this study demonstrates that covariates such as fine motor skill, baseline language, and other individual differences potentially attenuate gross motor relations over time with language during preschool and early childhood. Further work is necessary in this age range using longitudinal methods, as we caution drawing conclusion from a single study.

Wang et al. (2014) tested gross motor and language skills longitudinally, using a sample of Norwegian children followed at 3 and 5 years of age. Both gross motor and language skills were measured using the ASQ parent report questionnaire, which provides separate gross and fine motor scores, and a global language score. Correlations across time points for gross motor and language scores indicated that greater gross motor skill at 3 years was significantly positively correlated to higher language scores at both 3 and 5 years. However, when controlling for concurrent relations between gross motor, fine motor, language, and other demographic covariates, gross motor skills at 3 years did not predict language at 5 years. Analyses on concurrent gross motor and language relations that controlled for covariates did indicate that gross motor at 3 years was related to language at 3 years, and gross motor at 5 years was related to language at 5 years.

#### Synthesis of Gross Motor and Language Relations Across Infancy to Early Childhood

Overall, existing literature finds that gross motor skills demonstrate both concurrent and longitudinal relations with language skill across infancy, toddlerhood, preschool, and early childhood. A total of 15 articles found significant links between gross motor and language, even when accounting for other covariates. Thus, 75% of articles that assess gross motor and language relations published thus far report significant findings for gross motor. Interestingly, 100% of cross-sectional studies during preschool and early childhood, and 89% of longitudinal studies with infant and toddler samples reported significant relations between gross motor and language. In particular, measuring the onset of specific gross motor skills during infancy such as sitting and walking has provided powerful evidence demonstrating that experience in new postures and locomotion styles can predict receptive and expressive language at single time points, and growth over time (Walle and Campos, 2014; Libertus and Violi, 2016; West et al., 2017). Frequently, gross motor skills have been found to predict language ability above and beyond other factors such as age, general locomotion experience, SES, or parental influences (e.g., He et al., 2015; Muluk et al., 2016; Walle, 2016). However, global scores from standardized assessments have also provided insight on gross motor skills and language relations, but have sometimes not found significant relations to language longitudinally (Wang et al., 2014; Leonard et al., 2015). Changes in the predictive capacity of gross motor skills over time is particularly clear as gross motor and language relations are explored at older ages closer to preschool entry (Cameron et al., 2012; Oudgenoeg-Paz et al., 2016). Importantly, it is possible that the inconsistency in gross motor to language relations seen at older ages simply demonstrates that cascading effects from motor to language are limited in time. Behaviors such as walking may no longer foster the same level of growth in language once the behavior is no longer novel and the infant system is not in the process of learning a new skill (e.g., Oudgenoeg-Paz et al., 2016). While cross-sectional studies during the ages spanning preschool and early childhood have found relations between gross motor and language, studies focusing on outcomes over time find mixed results, with gross motor prior to kindergarten predicting expressive language skills in Spring of kindergarten, but studies with time points further apart demonstrating less of an influence of earlier motor skill on later language (Cameron et al., 2012; Wang et al., 2014). In terms of quantity however, more studies have been conducted during infancy and toddlerhood on the relation between gross motor and language compared to early childhood, which limits our interpretation of findings for the older age ranges.

### Fine Motor Skills and Language Development

The following section will provide existing evidence regarding the relation between fine motor skills and language outcomes. Some of the studies reported in this section are the same studies from the gross motor skills and language development section, as multiple studies included in this review measured both gross and fine motor skills. Here, results will only focus on fine motor measures and language of these articles. A synthesis of all studies included in the fine motor skills and language development section will be provided at the end of this section.

#### Cross-Sectional Studies With Infants and Toddlers

There are only two studies in the current review that utilized cross-sectional samples to analyze fine motor skills in relation to language development in infancy and toddlerhood (Alcock and Krawczyk, 2010; Houwen et al., 2016). Results reviewed here are based on UK and Dutch infants. One study utilized standardized assessments to measure both fine motor and language skills (Houwen et al., 2016), and the other study used a combination of standardized assessments and parent report (Alcock and Krawczyk, 2010). Both studies find at least one link between fine motor skills and receptive and productive language prior to analyses with covariates. Both studies utilized covariates, with Houwen et al. (2016) indicating that fine motor skills continued to predict language skills after controlling for cognitive levels. In comparison, Alcock and Krawczyk (2010) found that when controlling for numerous covariates such as gross motor skill, oral motor skill, and gesture among other variables, fine motor skills were no longer related to language skills. Overall, the set of cross-sectional studies on fine motor skills and language reviewed below demonstrate that concurrent relations do exist between fine motor and language, but highlight that this relation may sometimes be explained via other variables. However, too few cross-sectional studies are available at this age range to make concrete conclusions regarding concurrent relations between fine motor and language.

Studying children across 3 months to 3 years using the BSID to measure fine motor and receptive and expressive language, Houwen et al. (2016) found that fine motor skills were significantly positively correlated with expressive and receptive communication scores, above and beyond cognitive level. Alcock and Krawczyk (2010) measured fine motor skills across two subsets of children at 21 months of age using the BSDI or an adapted questionnaire that included fine motor questions from the ASQ parent report questionnaire. Language skills assessed using the Oxford MCDI with additional questions on from the U.S. English MCDI concerning word combinations and grammatical usage (e.g., complexity). Fine motor scores based on parent report were significantly positively correlated to receptive and expressive vocabulary, but not complexity. When standard scores were used to combine parent reported fine motor scores and BSDI scores, a significant and positive correlation was found for fine motor skill and receptive and expressive vocabulary, but not complexity. Neither standardized fine motor scores or fine motor questionnaire scores alone were significantly related to receptive, expressive vocabulary, or language complexity when accounting for oral movement, gross motor score, gesture, and symbolic gesture, among other control variables.

#### Longitudinal Studies With Infants and Toddlers

Six studies measured longitudinal relations between fine motor skills and language outcomes across infancy and toddlerhood (Butterworth and Morissette, 1996; Lyytinen et al., 2001; Leonard et al., 2015; Oudgenoeg-Paz et al., 2015; Libertus and Violi, 2016; Choi et al., 2018). Samples reported on here include U.S., Dutch, and Finnish infants. The majority of the studies reported here (five out of six) measured fine motor and language skills via parent report or in lab measures, with only one study utilizing a standardized measures (Choi et al., 2018). Only two studies (about 34%) found a significant relation between fine motor skill at an early time point and later language outcomes (Lyytinen et al., 2001; Choi et al., 2018). However, both studies do not share much communality in methodology: one study found cascading effects of fine motor skills at 6 months to later language at 36 months, indicating that fine motor skills measured based on standardized assessment can have a cascading relation to language development over a 30 month time span (Choi et al., 2018). The second study assessed fine motor ability based on infant deviation from the median growth curve of fine motor skill milestones and used parent reported language at 12, 14, and 24 months (Lyytinen et al., 2001). Measures across both studies differed, as did the ages assessed. Choi et al. (2018) did however control for visual reception skills among other demographic covariates and continued to find a significant link between fine motor and later language, which supports the idea that fine motor skills predict language beyond general cognitive skills. More detailed summaries for this set of studies are included below.

Using parent reported onset of fine motor skills and the MCDI: WG as a measure of language skills, Lyytinen et al. (2001) found that infant's deviation from a calculated median growth curve based on fine motor skill milestone attainment over the first year of life was predictive of vocabulary comprehension at 12 and 14 months, and vocabulary production at 14 and 30 months (but not production at 24 months). Libertus and Violi (2016) measured longitudinal changes in grasping ability from 3 to 5 months of age, and measured language using the MCDI: WG at 10 and 14 months. Findings indicated that growth in grasping duration was not significantly correlated with receptive vocabulary at 10 and 14 months of age.

Similarly, Choi et al. (2018) also measured growth in fine motor skill in typically developing infants and in a sample of infants at high risk for ASD. Using the MSEL fine motor subscale, fine motor skills were measured from 6 to 24 months every 6 months. Expressive language skill was measured at 36 months using the MSEL expressive language subscale. For typically developing infants, high levels of fine motor skill at 6 months was predictive of greater expressive language scores at 36 months, while controlling for visual receptive skills, sex, and SES. Linear growth and quadratic growth in fine motor skills were not predictive of language scores at 36 months while accounting for covariates. Comparably, when measuring fine motor skills at 7 months using the MSEL, and receptive and expressive language at 7, 14, 24, and 36 months using the Vineland Adaptive Behavior Scales, Leonard et al. (2015) found that fine motor skills were not predictive of receptive or expressive language growth while controlling for visual-reception skills.

A study by Butterworth and Morissette (1996) measured pincer grip skills monthly from 8.5 to 14.5 months of age. Language was also measured monthly using the MCDI: WG. Pincer grip onset was not significantly related to MCDI comprehension or production scores at 14.5 months. Measuring fine motor skills and language later, Oudgenoeg-Paz et al. (2015) observed exploration through relational object exploration in lab at 20 months, and assessed production of spatial language at 36 months based on two in lab tests. Results indicated that duration of spatial relational object exploration at 20 months was not related to spatial language at 36 months.

#### Cross-Sectional Studies Spanning Pre-kindergarten and Early Childhood

A total of six studies assessed the relation between fine motor skills and language during early childhood and preschool age using cross-sectional methods and analyses (Wolff and Wolff, 1972; Iverson and Braddock, 2010; Rhemtulla and Tucker-Drob, 2011; Cameron et al., 2012; Muluk et al., 2014; Suggate and Stoeger, 2014). Samples discussed here include U.S., German, and Turkish children. Four out of the six studies (about 67%) found significant relations between fine motor ability and language skills. Two studies calculated composite scores or a factor for fine motor skills based on actions observed in lab (Rhemtulla and Tucker-Drob, 2011; Suggate and Stoeger, 2014), one study created a composite score from parent a parent report questionnaire and an in lab standardized assessment (Iverson and Braddock, 2010), one study used teacher report to measure fine motor skills (Wolff and Wolff, 1972), and another study utilized individual items drawn from a standardized assessment (Muluk et al., 2014). Cameron et al. (2012) measured fine motor skills using a standardized assessment, but used both a global score and individual items from the larger assessment to investigate links between fine motor and language. When measuring language skills, one study created a composite score from in lab observations and a standardized assessment (Iverson and Braddock, 2010), one study used items derived from a standardized assessment (Muluk et al., 2014), one used in lab observation exclusively (Rhemtulla and Tucker-Drob, 2011), and one only used a standardized assessment for language (Cameron et al., 2012). Suggate and Stoeger (2014) used a standardized assessment to measure receptive language skills, but also measured receptive vocabulary regarding body related objects and actions to test potential links between fine motor and language via the concept of embodiment. Four studies included covariates, with two of these studies demonstrating continued relations between fine motor and language while accounting for variability in other domains (Muluk et al., 2014; Suggate and Stoeger, 2014). In general, results in this section indicate that fine motor skills are concurrently related to language ability during preschool age and early childhood.

In a sample of typically developing children and children with language impairment ranging from 3 to 5 years old, Iverson and Braddock (2010) measured fine motor skills using the Child Development Inventory parent report instrument and the Battelle Developmental Screening Inventory. Language skills were measured using the PLS and also measures of verbal utterances per minute, number of different words used, and mean length of utterance were generated from a 10 min in lab observation. A single composite score was created for fine motor and another composite score for language skills. Results indicated that for the typical group, fine motor was not predictive of language skills when including gesture skills as a covariate.

In their cross-sectional study, Muluk et al. (2014) provided separate correlations and analyses for children 3 to 6 years old with results of interest for the current review including only 3 to 5 years. Fine motor and language skills were measured using individual items from the Denver II adapted for use in Turkey. At 3 years, the fine motor skill of “imitating a vertical line” was positively significantly correlated with the language skills of “using plurals,” “defining six words,” and being able to “give first and last name.” However, these relations were no longer significant when accounting for a host of covariates determined via backwards regression. The ability to “imitate a bridge” was significantly positively correlated with the ability to “use plurals,” “name three pictures,” “point to four pictures,” “produce fully understandable speech,” “define six words,” and being able to “give first and last name.” However, when controlling for various covariates, the ability to imitate a bridge was significantly related to “using plurals,” “naming three pictures,” and being able to “give first and last name.” The ability to “build a tower of 7 blocks” was significantly positively correlated with language skills such as “knowing one function,” and “being able to define six words,” but these relations were no longer significant when accounting for various covariates. At 4 years, the ability to “copy a circle” was significantly positively correlated to language skills such as “knowing the use of one object,” but was not significant when accounting for other covariates during backwards regression analyses. At 5 years, being able to copy a circle, cross, and a square were all significantly positively correlated with being able to “define six words,” and “counting two blocks.” Being able to “draw a man” was significantly positively correlated with “defining six words,” “counting two blocks,” and being able to “tell opposites.” Copying a cross continued to be significantly related to “defining six words,” and “drawing a man” also continued to be significantly related to being able to “tell opposites” when accounting for various other covariates.

Suggate and Stoeger (2014) also measured fine motor skills and language development during preschool age. Fine motor skills were measured using 3 tasks: pegboard task, peg threading, and block turning. A single factor was created for fine motor skills. General receptive language skills were measured using the German adaptation of the PPVT. This study was specifically interested in words with high levels of body-object interaction (e.g., belt; BOI), so and additional measure of BOI receptive vocabulary based on words selected from the PPVT was used as well. Receptive vocabulary for words that pertain to referents that are easily manually manipulated were also selected from the PPTV as a separate language measure. Fine motor skills were significantly positively correlated with general vocabulary, BOI vocabulary, and manipulable vocabulary, even when controlling for age. Mediation analyses suggested that BOI vocabulary significantly mediated the relation between both general and manipulation vocabulary and fine motor skill. Using exclusively teacher report measures, Wolff and Wolff (1972) also assessed the relation between fine motor and language skills. Fine motor skills were significantly positively related to both verbal output and verbal skill scores.

A longitudinal study by Rhemtulla and Tucker-Drob (2011) provided separate cross-sectional data regarding fine motor skills and language outcomes at 4 years. Fine motor skills as measured in lab by experimenters based on activities such as building a gate from wooden blocks after watching an experimenter build it out of a second set of blocks, and copying three shapes (a square, a triangle, and an asterisk) with a composite score calculated from all activities. Oral language skills were also measured using the Lets Tell Stories task. Results indicated that oral language skills at 4 years were significantly positively correlated to fine motor scores measured concurrently.

In their study investigating the relation between fine motor skills prior to kindergarten entry and language in kindergarten, Cameron et al. (2012) used the Early Screening-Inventory-Revised to measure fine motor skills and the Woodcock Johnson Vocabulary subtest to measure language production. Although the Early Screening-Inventory-Revised provides a composite fine motor score, Cameron et al. (2012) also used the individual fine motor items (block use, design copy, and drawing-a-person) when analyzing fine motor and language relations. The fine motor composite was significantly positively correlated with expressive vocabulary in fall of kindergarten. Specifically, block use was significantly positively correlated with fall expressive language, while design copy skills were not significantly correlated to fall expressive vocabulary. The ability to Draw-a-Person was not correlated to expressive language. However, fine motor skills did not predict expressive language skill above and beyond gross motor skills, or other covariates such as executive functioning, age, sex, ethnicity, maternal education, or age at motor assessment.

#### Longitudinal Studies Spanning Pre-kindergarten and Early Childhood

One study selected for this systematic review examined the relation between fine motor skills and language outcomes longitudinally spanning preschool age and early childhood (Wang et al., 2014). Wang et al. (2014) used an established parent questionnaire to measure both fine motor and language skills. Analyses utilized covariates, with results indicating that longitudinal fine motor and language links may potentially be explained via other variables. However, more work is needed to draw stronger conclusions regarding longitudinal links between fine motor and language skills during preschool and early childhood given the limited amount of studies available.

Results from Wang et al. (2014) demonstrated that fine motor skills at 3 years were correlated to language at 5 years, but not when accounting for Apgar score, birthweight, gestational age, parent's age, education, income, native language, and maternal psychological distress, and fine motor and language scores at 3 years. Fine motor skills at 3 years were significantly related to concurrent language skill at 3 years (even when accounting for covariates). Similarly, fine motor skills at 5 years were significantly related to language at 5 years, while controlling for covariates. Fine motor and a global language scores from the ASQ were used for this study.

#### Synthesis of Fine Motor and Language Relations Across Infancy to Early Childhood

Overall, studies measuring fine motor and language relations demonstrate mixed findings. Of the 15 studies total that measured fine motor skills, only 8 found that fine motor skill was significantly related to language outcomes. This pattern indicates that currently only about 53% of articles that measure fine motor skills demonstrate a significant relation with language outcomes. The most consistent findings originate from cross-sectional studies during preschool and early childhood, where about 67% of studies found significant relations between fine motor and language. Concurrent links between fine motor and language are also supported in this age group by Wang et al. (2014), who found in their longitudinal study that fine motor skills and language ability were related within time points, but fine motor skills at 3 years did not predict language at 5 years. Choi et al. (2018) did find longitudinal relations between fine motor and language, with fine motor skills at 6 months of age predicting expressive language skills at 3 years old. Similarly, Lyytinen et al. (2001) also demonstrate that fine motor skills relate to language in infancy and toddlerhood.

However, fine motor skills have been measured less than gross motor in the current literature (15 fine motor inclusive articles vs. 20 gross motor inclusive articles). In order to more thoroughly conclude whether gross motor or fine motor skills provide a better predictor for language outcomes, the final section of the results will compare results from studies that measured both gross and fine motor skills together, and assess the frequency fine motor and gross motor were found to significantly predict language outcomes from this subset of articles.

### Concurrent Measurement of Gross Motor vs. Fine Motor Skills

Eleven studies included in the current systematic review measured both gross motor and fine motor skills (Wolff and Wolff, 1972; Lyytinen et al., 2001; Alcock and Krawczyk, 2010; Rhemtulla and Tucker-Drob, 2011; Cameron et al., 2012; Muluk et al., 2014; Wang et al., 2014; Leonard et al., 2015; Oudgenoeg-Paz et al., 2015; Houwen et al., 2016; Libertus and Violi, 2016). Five studies were cross-sectional (Wolff and Wolff, 1972; Alcock and Krawczyk, 2010; Rhemtulla and Tucker-Drob, 2011; Muluk et al., 2014; Houwen et al., 2016) and six studies were longitudinal (Lyytinen et al., 2001; Cameron et al., 2012; Wang et al., 2014; Leonard et al., 2015; Oudgenoeg-Paz et al., 2015; Libertus and Violi, 2016). Six studies spanned infancy and toddlerhood (Lyytinen et al., 2001; Alcock and Krawczyk, 2010; Leonard et al., 2015; Oudgenoeg-Paz et al., 2015; Houwen et al., 2016; Libertus and Violi, 2016), and five studies were based on samples of children at preschool age and in early childhood (Wolff and Wolff, 1972; Rhemtulla and Tucker-Drob, 2011; Cameron et al., 2012; Muluk et al., 2014; Wang et al., 2014).

When focusing on only studies that measure both gross motor and fine motor, fine motor skills demonstrate a higher frequency of significant findings than gross motor skills. Three studies find that fine motor skills relate to language outcomes more frequently than gross motor skills (Wolff and Wolff, 1972; Lyytinen et al., 2001; Houwen et al., 2016). Houwen et al. (2016) found that fine motor scores were significantly positively correlated to expressive and receptive language above and beyond cognitive level in a cross-sectional sample with infants from 3 months to 3 years. Gross motor scores were not positively correlated to language while accounting for cognitive level. Lyytinen et al. (2001) also found that fine motor skills were significantly correlated to language at more time points than gross motor. Fine motor skills were significantly correlated at 12 and 14 months with vocabulary comprehension, and vocabulary production at 14 and 30 months, while gross motor skill was only significantly correlated with vocabulary comprehension at 12 and 14 months, but not with productive vocabulary at any time point across 14, 24, and 30 months. During preschool, Wolff and Wolff (1972) similarly found that fine motor skills were concurrently related to both verbal output and verbal quality, while gross motor skills were only correlated with verbal output. Two studies found that gross motor skills predicted language outcomes more frequently than fine motor skills (Oudgenoeg-Paz et al., 2015; Libertus and Violi, 2016).

However, three studies also found that both gross and fine motor skills are significantly predictive of language skills with similar frequency (Rhemtulla and Tucker-Drob, 2011; Muluk et al., 2014; Wang et al., 2014). In the case of Muluk et al. (2014), use of multiple individual behaviors to measure gross, fine motor, and language skills revealed three gross motor skills were predictive of five language skills across 3 to 5 years, and three fine motor skills that were predictive of five language skills as well. Rhemtulla and Tucker-Drob (2011) found that both gross motor and fine motor skills were correlated to oral language skills. To further attempt to disentangle these results, a more detailed focus on effect sizes finds that the correlation coefficient for fine motor skills and language was 0.32 and the correlation coefficient for gross motor skill and language was 0.29, indicating that both results had roughly a medium effect. For the study by Wang et al. (2014), both gross motor and fine motor scores were correlated with language skills at concurrent time points (3 and 5 years of age), but not longitudinally. Effect sizes for gross motor skill and language were 0.56 and 0.35 for 3 and 5 years respectively, and 0.44 and 0.34 for fine motor skill. Comparably however, three studies also found that neither gross motor skill or fine motor skill predict language abilities when accounting for additional covariates (Alcock and Krawczyk, 2010; Cameron et al., 2012; Leonard et al., 2015). Overall, when limiting findings to studies that measure both gross and fine motor skills for comparison between the two skill types, frequency of significant findings are closely balanced, with fine motor skills demonstrating a slight edge on gross motor skills by only one study.

Overall, we find that both gross and fine motor are related to language outcomes. However, given the low frequency of fine motor research in relation to language, no conclusions can be drawn at the moment regarding whether one skill is more closely related to language than the other.

## Discussion

The current systematic review assessed existing literature on the relation between motor and language development, and aimed to discern whether gross or fine motor skills predicted language skills more frequently. Given the available studies to draw from, a main take away from this systematic review is that both gross and fine motor skills help foster language development. However, fine motor skills have been less studied in relation to language. Thus, we caution against claiming that one motor skill type is more important than the other.

Our conclusion that both gross and fine motor skills matter for language does not mean that both motor skill types provide for language development via the same mechanisms. Although focusing on mechanism was not a goal of the current review, it is important to note that it is likely that gross and fine motor development may support language via different means. Gross motor skills such as crawling and walking allow infants to travel independently throughout their immediate environments, traversing long distances to encounter objects and caregivers. However, even within these two skills that seemingly provide the same advantage (locomotion), infants are in widely different postures, which reframes what infants are able to observe (Kretch et al., 2014). Similarly, fine motor skills such as grasping and drawing are both related as they fall under the same motor skill umbrella, but may provide very different affordances for language learning. Recent work in infants has begun to explore potential mechanisms that underlie motor-language links (Walle, 2016; West and Iverson, 2017; McQuillan et al., 2019), but further research is needed to better understand what it is about motor skills, both gross and fine, that fosters language development.

The length of motor-language cascades was a common theme of the systematic review results. Both gross motor and fine motor skills demonstrated longitudinal effects toward later language outcomes (Lyytinen et al., 2001; Libertus and Violi, 2016; West et al., 2017; Choi et al., 2018). However, some findings indicate that the length of these cascades are limited, or perhaps even constrained to concurrent relations depending on the age range (Wang et al., 2014; Oudgenoeg-Paz et al., 2016). We speculate that the temporal frame in which a motor skill is measured in relation to language likely matters for finding relations depending on the age of interest. For example, Oudgenoeg-Paz et al. (2016) no longer find that age of walking acquisition predicts spatial language at 43 months, but exploration via self-locomotion measured at 20 months does predict later spatial language. For fine motor skills, the majority of findings that indicate a relation between fine motor and language are based on analyses of concurrent fine motor and language measurements, which may indicate that fine motor measures used in existing longitudinal studies may not fully tap into the appropriate fine motor skill at the appropriate age.

Common themes can be drawn however based on skills most commonly measured in the literature. In the case of gross motor skills, walking is the most frequent motor measure. Commonly, walking onset has been used on its own to measure length of motor experience (e.g., Oudgenoeg-Paz et al., 2012, 2015, 2016; West et al., 2017) or in comparison to crawling (e.g., Karasik et al., 2014; Walle and Campos, 2014; He et al., 2015; Walle, 2016). Overall, findings based solely on walking overwhelmingly indicate that walking is an important phenomenon tied to language outcomes. Particularly, walking is important for infant vocabulary, the most common measure of language in this area of research. In terms of fine motor skills, there is greater variability in measurement type, making a firmer conclusion difficult to reach. The variability in fine motor measures in the studies discussed in the current systematic review may have contributed to conflicting results regarding the relation between fine motor skills and language.

Fairly, it is possible that the smaller number of studies on fine motor skills and language seen in this review, and the variability seen in these few studies, stems from a lack of a “holy grail” fine motor measure from 0 to 5 years of age. Gross motor measures were mostly based on parent report, which included report of motor milestones such as sitting and walking Fine motor skills are arguably hidden in plain sight during what many would label as play: opening a box, learning to use a marker to draw, or playing with blocks. It is imperative that researchers interested in motor development begin to consider fine motor skills potentially from a milestone perspective. Researchers need not look far to find potential fine motor skills that could fit milestone criteria, as research on handedness provides a rich literature on measuring development in skills such as grasping, unimanual manipulation, and role differentiated bimanual manipulation, the latter of which continues to be a challenging fine motor skill across infancy to early childhood (Michel et al., 2013; Nelson et al., 2013, 2018; Campbell et al., 2015). Critically, longitudinal research finds that consistency in hand preference for fine motor skills such as role differentiated bimanual manipulation across toddlerhood is predictive of language outcomes at 2 and 3 years of age (Nelson et al., 2014, 2017). Thus, it may be possible that long-term consistency in hand use in early development captures a greater level of fine motor skill, in turn supporting language development; however the relation between consistency and fine motor skill remains to be explicitly tested. Overall, researchers interested in motor-language cascades should be aware that a lack of consensus in the field regarding how fine motor skills are measured may underlie the variability in results regarding fine motor skills relating to language outcomes.

Language development has long captivated researchers, and with good reason: language allows our species to communicate with one another in ways that other forms of communication may not readily provide (Corballis, 2009). But just as memorable as children's first words are their first steps and the time they draw their first scribbles. Motor development has for several decades provided researchers with the ability to measure and quantify behavior, with motor skills often playing a central but quiet role in some of our field's most important research paradigms and findings (e.g., Piaget, 1954; Rovee-Collier et al., 1980; Walk and Gibson, 2011). As evidenced by the current systematic review, a recent revival has occurred in bringing motor development back into the fold of cognition (Rosenbaum, 2005; Adolph et al., 2010; Iverson, 2010). We hope that researchers embrace motor skills, gross and fine, as important toward our understanding of language development.

## Author Contributions

SG and EN conceptualized the idea of conducting a systematic review. SG conducted the initial article search across databases. SG, VA, and EN discussed final inclusion and exclusion criteria. SG and VA reviewed abstracts and articles for inclusion in the current systematic review. VA provided a first draft of Table 1, Figure 1, and Supplementary Table 1, with the versions included in the current manuscript finalized by SG. SG wrote the first draft of the manuscript. VA and EN provided additional comments and edits on the manuscript, tables and figures. All authors contributed to and approved the final manuscript.

### Conflict of Interest

The authors declare that the research was conducted in the absence of any commercial or financial relationships that could be construed as a potential conflict of interest.
