# Supplementary material for: Do Gross and Fine Motor Skills Differentially Contribute to Language Outcomes? A Systematic Review
Source: Front Psychol. 2019 Dec 3;10:2670. doi: 10.3389/fpsyg.2019.02670 (PMC6901663; doi:10.3389/fpsyg.2019.02670)
Supplement: Supplementary file 1 [file Table_1.docx]

Supplementary Material

**Supplementary Table 1. Quality assessment of included peer-reviewed articles**

|  |  | Alcock & Krawczyk (2010) | Butterworth & Morissette (1996) | Cameron et. al (2012) | Choi et. al (2018) | He, Walle & Campos (2015) | Houwen et. al (2016) |
| --- | --- | --- | --- | --- | --- | --- | --- |
| Introduction | Were the aims/objectives of the study clear? | Yes | Yes | Yes | Yes | Yes | Yes |
| Methods | Was the study design appropriate for the stated aim(s)? | Yes | Yes | Yes | Yes | Yes | Yes |
|  | Was the sample size justified? | No | No | No | No | No | Yes |
|  | Was the target/reference population clearly defined? (Is it clear who the research was about) | Yes | Yes | Yes | Yes | Yes | Yes |
|  | Was the sample frame taken from an appropriate population base so that it closely represented the target/reference population under investigation? | Yes | No | Yes | Yes | Yes | Yes |
|  | Was the selection process likely to select subjects/participants that were representative of the target/reference population under investigation? | Yes | Yes | Yes | Yes | Yes | Yes |
|  | Were measures undertaken to address non-responders? | No | No | Yes | Yes | No | No |
|  | Were the independent and dependent variables measured appropriate to the aims of the study? | Yes | Yes | Yes | Yes | Yes | Yes |
|  | Were the independent and dependent variables measured correctly using instruments/ measurements that had been trialed, piloted or published previously? | Yes | Yes | Yes | Yes | Yes | Yes |
|  | Is it clear what was used to determined statistical significance and/or precision estimates? (e.g., p values, CIs) | Yes | Yes | Yes | Yes | Yes | Yes |
|  | Were the methods (including statistical methods) sufficiently described to enable them to be repeated? | Yes | Yes | Yes | Yes | Yes | Yes |
| Results | Were the basic data adequately described? | Yes | Yes | Yes | Yes | Yes | Yes |
|  | Does the response rate raise concerns about non-response bias? | No | No | No | No | No | No |
|  | If appropriate, was information about non-responders described? | Yes | Yes | Yes | Yes | Yes | No |
|  | Were the results internally consistent? | Yes | Yes | Yes | No | Yes | Yes |
|  | Were results for analyses described in the methods presented? | Yes | Yes | Yes | Yes | Yes | Yes |
| Discussion | Were the authors’ discussions and conclusions justified by the results? | Yes | Yes | Yes | Yes | Yes | Yes |
|  | Were the limitations of the study discussed? | Yes | No | Yes | Yes | Yes | Yes |
| Other | Were there any funding or conflicts of interest that may affect result interpretation? | No | No | No | No | No | N/A |
|  | Was ethical approval attained? | N/A | N/A | N/A | Yes | N/A | Yes |
|  | Comments | Ethics statement not included | Ethics statement not included | *Label as cross-sectional based on results reported for systematic review; Ethics statement not included |  | Ethics statement not included; discuss missing data but unclear what actions were taken | Funding/Conflict of Interest statement not included |

|  |  | Iverson & Braddock (2010) | Karasik, Tamis-LeMonda & Adolph (2014) | Leonard, Bedford & Pickles (2015) | Libertus & Violi (2016) | Lyytinen et. al (2001) | Muluk, Bayoǧlu, & Anlar (2014) |
| --- | --- | --- | --- | --- | --- | --- | --- |
| Introduction | Were the aims/objectives of the study clear? | Yes | Yes | Yes | Yes | Yes | Yes |
| Methods | Was the study design appropriate for the stated aim(s)? | Yes | Yes | Yes | Yes | Yes | Yes |
|  | Was the sample size justified? | No | No | No | No | No | Yes |
|  | Was the target/reference population clearly defined? (Is it clear who the research was about) | Yes | Yes | Yes | Yes | Yes | Yes |
|  | Was the sample frame taken from an appropriate population base so that it closely represented the target/reference population under investigation? | Yes | Yes | Yes | Yes | Yes | Yes |
|  | Was the selection process likely to select subjects/participants that were representative of the target/reference population under investigation? | Yes | Yes | Yes | Yes | Yes | Yes |
|  | Were measures undertaken to address non-responders? | No | Unclear | Unclear | No | Unclear | Unclear |
|  | Were the independent and dependent variables measured appropriate to the aims of the study? | Yes | Yes | Yes | Yes | Yes | Yes |
|  | Were the independent and dependent variables measured correctly using instruments/ measurements that had been trialed, piloted or published previously? | Yes | Yes | Yes | Yes | Yes | Yes |
|  | Is it clear what was used to determined statistical significance and/or precision estimates? (e.g., p values, CIs) | Yes | Yes | Yes | Yes | Yes | Yes |
|  | Were the methods (including statistical methods) sufficiently described to enable them to be repeated? | Yes | Yes | Yes | Yes | Yes | Yes |
| Results | Were the basic data adequately described? | Yes | Yes | Yes | Yes | Yes | Yes |
|  | Does the response rate raise concerns about non-response bias? | No | N/A | N/A | No | N/A | N/A |
|  | If appropriate, was information about non-responders described? | Yes | N/A | N/A | Yes | N/A | N/A |
|  | Were the results internally consistent? | Yes | Yes | Yes | Yes | Yes | Yes |
|  | Were results for analyses described in the methods presented? | Yes | Yes | Yes | Yes | Yes | Yes |
| Discussion | Were the authors’ discussions and conclusions justified by the results? | Yes | Yes | Yes | Yes | Yes | Yes |
|  | Were the limitations of the study discussed? | Yes | Yes | Yes | Yes | No | No |
| Other | Were there any funding or conflicts of interest that may affect result interpretation? | No | No | No | No | No | No |
|  | Was ethical approval attained? | N/A | N/A | N/A | Yes | N/A | N/A |
|  | Comments | Ethics statement not included | Unclear if there were any non-responders; Ethics statement not included | Unclear if there were any non-responders; Ethics statement not included |  | Unclear if there were any non-responders; Ethics statement not included | Unclear if there were any non-responders; Ethics statement not included |

|  |  | Muluk, Bayoǧlu, & Anlar (2016) | Oudgenoeg-Paz, Volam, Lesemam (2012) | Oudgenoeg-Paz, Volam, Lesemam (2015) | Oudgenoeg-Paz, Volam, Lesemam (2016) | Rhemtulla & Tucker-Drob (2011) | Suggate & Stoeger (2014) |
| --- | --- | --- | --- | --- | --- | --- | --- |
| Introduction | Were the aims/objectives of the study clear? | Yes | Yes | Yes | Yes | Yes | Yes |
| Methods | Was the study design appropriate for the stated aim(s)? | Yes | Yes | Yes | Yes | Yes | Yes |
|  | Was the sample size justified? | No | No | No | No | No | No |
|  | Was the target/reference population clearly defined? (Is it clear who the research was about) | Yes | Yes | Yes | Yes | Yes | Yes |
|  | Was the sample frame taken from an appropriate population base so that it closely represented the target/reference population under investigation? | Yes | Yes | Yes | Yes | Yes | Yes |
|  | Was the selection process likely to select subjects/participants that were representative of the target/reference population under investigation? | Yes | Yes | Yes | Yes | Yes | Yes |
|  | Were measures undertaken to address non-responders? | Unclear | Yes | Yes | Yes | Yes | Yes |
|  | Were the independent and dependent variables measured appropriate to the aims of the study? | Yes | Yes | Yes | Yes | Yes | Yes |
|  | Were the independent and dependent variables measured correctly using instruments/ measurements that had been trialed, piloted or published previously? | Yes | Yes | Yes | Yes | Yes | Yes |
|  | Is it clear what was used to determined statistical significance and/or precision estimates? (e.g., p values, CIs) | Yes | Yes | Yes | Yes | Yes | Yes |
|  | Were the methods (including statistical methods) sufficiently described to enable them to be repeated? | Yes | Yes | Yes | Yes | Yes | Yes |
| Results | Were the basic data adequately described? | No | Yes | Yes | Yes | Yes | Yes |
|  | Does the response rate raise concerns about non-response bias? | N/A | No | No | No | No | No |
|  | If appropriate, was information about non-responders described? | N/A | No | Yes | Yes | Yes | Yes |
|  | Were the results internally consistent? | Yes | Yes | Yes | Yes | Yes | Yes |
|  | Were results for analyses described in the methods presented? | No | Yes | Yes | Yes | Yes | Yes |
| Discussion | Were the authors’ discussions and conclusions justified by the results? | Yes | Yes | Yes | Yes | Yes | Yes |
|  | Were the limitations of the study discussed? | No | Yes | Yes | Yes | No | Yes |
| Other | Were there any funding or conflicts of interest that may affect result interpretation? | No | No | N/A | No | No | No |
|  | Was ethical approval attained? | N/A | N/A | N/A | Yes | N/A | N/A |
|  | Comments | Do not mention non-responders; Did not provide full results for all variables; Ethics statement not included | Ethics statement not included | Funding/Conflict of interest statement not included; Ethics statement not included |  | *Label as cross-sectional based on results reported for systematic review; Ethics statement not included | Ethics statement not included |

|  |  | Walle (2016) | Walle & Campos (2014) | Wang et. al (2014) | West et. al (2017) | Wolff & Wolff et. al (1972) |
| --- | --- | --- | --- | --- | --- | --- |
| Introduction | Were the aims/objectives of the study clear? | Yes | Yes | Yes | Yes | Yes |
| Methods | Was the study design appropriate for the stated aim(s)? | Yes | Yes | Yes | Yes | Yes |
|  | Was the sample size justified? | No | No | No | No | No |
|  | Was the target/reference population clearly defined? (Is it clear who the research was about) | Yes | Yes | Yes | Yes | Yes |
|  | Was the sample frame taken from an appropriate population base so that it closely represented the target/reference population under investigation? | Yes | Yes | Yes | Yes | Yes |
|  | Was the selection process likely to select subjects/participants that were representative of the target/reference population under investigation? | Yes | Yes | Yes | Yes | Yes |
|  | Were measures undertaken to address non-responders? | Yes | Yes | Yes | Yes | Unclear |
|  | Were the independent and dependent variables measured appropriate to the aims of the study? | Yes | Yes | Yes | Yes | Yes |
|  | Were the independent and dependent variables measured correctly using instruments/ measurements that had been trialed, piloted or published previously? | Yes | Yes | Yes | Yes | Yes |
|  | Is it clear what was used to determined statistical significance and/or precision estimates? (e.g., p values, CIs) | Yes | Yes | Yes | Yes | Yes |
|  | Were the methods (including statistical methods) sufficiently described to enable them to be repeated? | Yes | Yes | Yes | Yes | Yes |
| Results | Were the basic data adequately described? | Yes | Yes | Yes | Yes | Yes |
|  | Does the response rate raise concerns about non-response bias? | No | No | No | No | N/A |
|  | If appropriate, was information about non-responders described? | Yes | Yes | No | Yes | N/A |
|  | Were the results internally consistent? | Yes | Yes | Yes | Yes | Yes |
|  | Were results for analyses described in the methods presented? | Yes | Yes | Yes | Yes | Yes |
| Discussion | Were the authors’ discussions and conclusions justified by the results? | Yes | Yes | Yes | Yes | Yes |
|  | Were the limitations of the study discussed? | Yes | Yes | Yes | Yes | No |
| Other | Were there any funding or conflicts of interest that may affect result interpretation? | No | N/A | No | No | No |
|  | Was ethical approval attained? | Yes | N/A | Yes | N/A | N/A |
|  | Comments |  | Funding/Conflict of interest statement not included; Ethics statement not included |  | Ethics statement not included | Unclear if there were any non-responders; Ethics statement not included |
